# Change of rhizospheric bacterial community of the ancient wild tea along elevational gradients in Ailao mountain, China

**DOI:** 10.1038/s41598-020-66173-9

**Published:** 2020-06-08

**Authors:** Haiyun Zi, Yonglei Jiang, Xiaomao Cheng, Wanting Li, Xiaoxia Huang

**Affiliations:** 1 Southwest Landscape Architecture Engineering Research Center of State Forestry and Grassland Administration, College of Landscape Architecture and Horticulture, Southwest Forestry University, Yunnan Kunming, 650224 China; 2 Yunnan Academy of Tobacco Agricultural Sciences, Kunming, 650021 China

**Keywords:** Soil microbiology, Environmental impact

## Abstract

The rhizospheric microbial community is one of the major environmental factors affecting the distribution and fitness of plants. Ancient wild tea plants are rare genetic resource distributed in Southwest China. In this study, we investigated that rhizospheric bacterial communities of ancient wild tea plants along the elevational gradients (2050, 2200, 2350 and 2500 m) in QianJiaZhai Reserve of Ailao Mountains. According to the Illumina MiSeq sequencing of 16 S rRNA gene amplicons, *Proteobacteria*, *Acidobacteria* and *Actinobacteria* were the dominant phyla with the relative abundance 43.12%, 21.61% and 14.84%, respectively. The *Variibacter* was the most dominant genus in rhizosphere of ancient wild tea plant. Phylogenetic null modeling analysis suggested that rhizospheric bacterial communities of ancient wild tea plants were more phylogenetically clustered than expected by chance. The bacterial community at 2050 m was unique with the highest alpha diversity, tend to cluster the nearest taxon and simple co-occurrence network structure. The unique bacterial community was correlated to multiple soil factors, and the content soil ammonium nitrogen (NH_4_^+^-N) was the key factor affecting the diversity and distribution of bacterial community along the elevational gradients. This study provided the necessary basic information for the protection of ancient tea trees and cultivation of tea plants.

## Introduction

The tea plant is an important economic crop in China. The QianJiaZhai Reserve is located in Yun-nan Ailao Mountains (Zhen-yuan County) with canopy coverage of more than 85%, where the ancient wild tea community, with an area of 280 hectares, is the largest community ever found in the world^1^. As the dominant plant species in this region, the diameter at breast height of these ancient tea plants are frequently more than 0.3 m, with probably more than 1000 years old. After thousands of years of growth, the ancient wild tea community in Qianjiazhai Reserve still maintains its large population and distributed in the elevation range (2100–2500 m). The enormous ancient tea community reflects the long history of tea cultivation and utilization by local nationalities, and its rich genetic resources are the material basis of germplasm innovation^2^. However, the ecological environment of ancient wild tea community has been affected by the population increases in the reserve region. The ancient wild tea communities are facing the challenge of habitat destruction, population decline and even extinction. Therefore, how to better protect and utilize the existing ancient wild tea resources is a large challenge.

The rhizosphere is the interface between plant roots and soil^3^, it plays a most direct influence on the soil for where the root grows and metabolic activities conduct. Within the rhizospheric soil, the bacterial community and its metabolic affect the nutrient transformation process such as the biogeochemical cycling of carbon, nitrogen, phosphorus, and other elements^4,5^. Moreover, the rhizospheric soil bacteria also provide indirect protection against pathogen or insect attack, thus positively influence plant growth and fitness^6–8^. In contrast, plant root exudates can lead to the variation of bacterial communities by either stimulating or inhibiting bacterial growth^9,10^. In addition, previous studies have suggested that rhizospheric bacterial communities were affected not only by the plant species, but also by some other factors, such as elevation, soil pH, and air humidity. For example, Debnath and colleagues^11^ have found that soil pH, total nitrogen, and organic matter had high correlations with the rhizobacterial community structure of medicinal plant *Rhododendron arboreum* at different elevations in Eastern slope of Himalayan Tawang region. Another study also revealed that the rhizobacterial of *Rhododendron arboretum* on the northern slope of Changbai Mountains was clearly influenced by soil pH value and content of nitrate nitrogen of soil^12^.

Recently, a few studies revealed the microbial community of tea rhizosphere and its interaction with environmental factors. For instance, Pandey and Palni^13^ found that species of *Bacillus* dominated the bacterial population in the established rhizosphere of tea bushes in the Himalayas region. Another report found that the dominant bacteria species of ancient tea tree is *Streptomyces*^14^. Research of Arafat^15^ on the south coast of Fujian province showed that the most dominant phyla in tea fields were *Proteobacteria* and *Acidobacteria*, and the bacterial taxa were affected by soil pH reduction during long-term tea cultivation. Besides soil pH, the soil organic carbon and available phosphorus meaningfully affected the rhizospheric microbial communities associated with long-term monoculture tea orchards was indicated by Li^16^. Most of these studies have documented the community assembly process and diversity of rhizospheric microbial communities in the well cultivated tea tree^17^, while, the rhizospheric microbiome studies on the endemic ancient wild plants, especially ancient wild tea community, are still largely unknown.

In our previous study, EST-SSR were used to study genetic diversity of ancient wild tea populations at different elevations in Qianjiazhai Reserve^18^. We found that the genetic characteristics of ancient wild tea were affected by the habitat heterogeneity in different elevation. Therefore, the elevational gradients of QianJiaZhai Reserve serve as ideal study areas^18^, for answering questions on how the rhizobacteria of the ancient wild tea community be affected by various environmental factors. In this study, we revealed the rhizospheric soil of ancient wild tea community along the gradient in QianJiaZhai Reserve of Ailao Mountains, China. We analyzed the physicochemical characteristics of rhizospheric soil, the rhizobacterial diversity and community composition of the ancient wild tea at four different elevations (2050, 2200, 2350 and 2500 m). Our results not only can provide the basic and necessary information to better understanding the ecological adaptation of ancient wild tea trees, but also can give theoretical reference for tea plant cultivation and germplasm resources conservation of wild ancient tea tree under the background of climate change and anthropogenic activities.

## Result

### Soil physiochemical properties

Elevation with 2050 m had the lowest values of total phosphorus (TP), available phosphorus (AP), soil organic matter (SOM), total carbon (C), total nitrogen (N), carbon/nitrogen ratio (C/N), ammonium nitrogen (NH^4+^-N) and nitrate nitrogen (NO^3^^–^N) compared with the other three higher elevations according to the One-way ANOVA test, and most of those edaphic factors had the highest values at 2500 m elevation except NH_4_^+^-N and NO_3_^–^N (Table 1). The value of NH_4_^+^-N is lowest at 2050 m (29.83 mg/kg), reaching a maximum at 2200 m (48.95 mg/kg), and then decreasing with elevation increased (Table 1). Similarly, the lowest and highest value of NO_3_^–^N was observed at 2350 m (14.25 mg/kg) and 2200 m (20.62 mg/kg), respectively (Table 1). The soil pH was acid in the sampled soil and varied from 4.56 to 4.64, there was no significant difference among the samples selected form different elevations.

**Table 1:** Physiochemical values of the rhizospheric soil in different elevations.

Indexes	Elevations (m)			
	2050	2200	2350	2500
TP (g/kg)	0.80 ± 0.10b	1.52 ± 0.06a	1.40 ± 0.09a	1.84 ± 0.28a
AP (g/kg)	0.19 ± 0.01b	0.20 ± 0.01ab	0.22 ± 0.01ab	0.24 ± 0.01a
SOM (g/kg)	70.44 ± 24.77c	157.29 ± 11.82ab	112.03 ± 24.22bc	203.78 ± 19.88a
N%	0.49 ± 0.06c	0.95 ± 0.05a	0.74 ± 0.06b	0.92 ± 0.09ab
C%	5.91 ± 0.86b	12.28 ± 0.63a	9.81 ± 0.82a	12.31 ± 1.37a
C/N ratio	11.84 ± 0.57b	12.95 ± 0.35ab	13.16 ± 0.27a	13.31 ± 0.27a
NH_4_^+^-N (mg/kg)	29.83 ± 1.07c	48.95 ± 2.11a	45.60 ± 3.02ab	39.84 ± 4.01b
NO_3_^–^N (mg/kg)	14.25 ± 0.89b	20.62 ± 2.02a	14.22 ± 0.44b	18.83 ± 1.85a
Soil moisture%	58.82 ± 6.94a	64.40 ± 6.13a	68.88 ± 2.60a	60.24 ± 2.25a
Soil pH	4.56 ± 0.04a	4.62 ± 0.37a	4.58 ± 0.37a	4.64 ± 0.60a

Data are indicated as mean ± standard error (SE), n = 5; diverse small letters ‘a’ and ‘b’ show the statistically significant difference in elevation gradients using one-way ANOVA tests (*P *< 0.05); TP, total phosphorus; AP, available phosphorus, SOM, soil organic matter; N, total nitrogen; C, total carbon; NH_4_^+^-N, ammonium nitrogen; NO_3_^–^N, nitrate nitrogen.

The value of TP, AP, SOM, N, C and C/N ratio presented a significantly positive relationship with elevation (*P* < 0.05), whereas no significant relationships with elevation detected on soil pH, NH_4_^+^-N, NO_3_^–^N or soil moisture (Table S1). Both NH_4_^+^-N and NO_3_^–^N were significantly correlated with organic nitrogen and carbon (*P* < 0.05). Moreover, the NH_4_^+^-N was positively correlated with C/N ratio while the NO_3_^–^N was positively correlated with TP (*P* < 0.05), respectively. In addition, the correlation results showed that there was a close relationship between TP and soil pH (*P* < 0.05) (Table S1).

### Changes in rhizospheric bacterial community composition and diversity

After a series of filtering, we obtained a total of 728,352 high-quality sequences from the 20 rhizospheric soil samples, ranging from 25,890 to 53,247 sequence per sample. A total of 1831 operational taxonomic units (OTUs) obtained across our samples were assigned to 24 bacterial phyla at a 97% sequence similarity cutoff.

**Figure 1:**
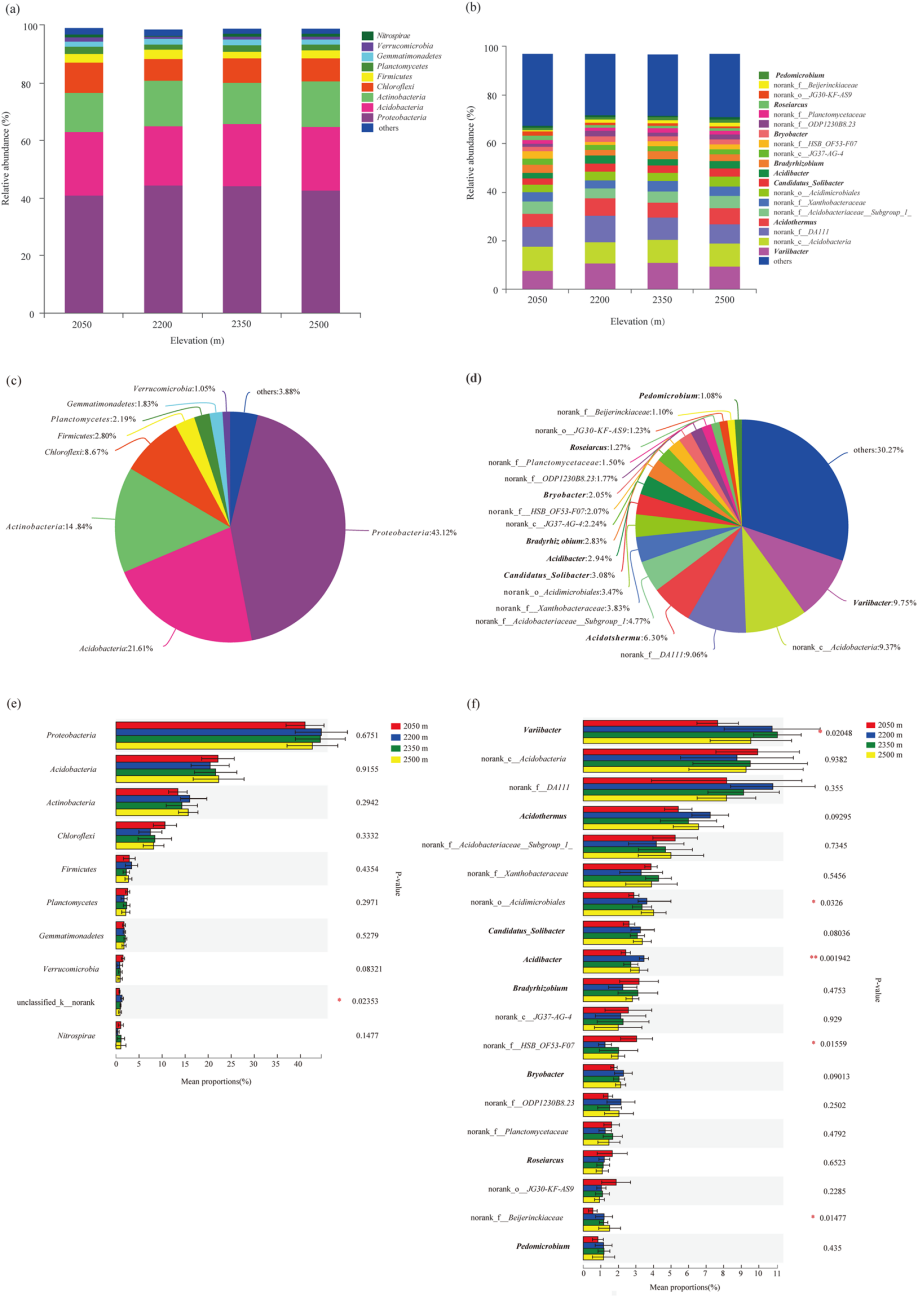
Classified summation of the relative abundance of bacterial phyla and genera in the different elevations. (**a,e**) The relative abundance of bacterial phyla in the different elevations; (**b**,**f**) The relative abundance of bacterial phyla in the different elevations; (**c**) Mean relative abundances of bacteria phyla in all samples. (**d**) Mean relative abundances of bacteria genus in all samples. The taxa are arranged as per total relative abundance across all samples, and only bacterial phyla (genera) with relative abundance≥1% was listed, and those with relative abundance<1% were summarized as others. The most abundant phyla (genera) at the bottom and the least abundant phyla (genera) at the top of the y-axis; Similarly, the phyla (genera) names in the legend are arranged from the most abundant at the bottom to the least abundant at the top.

In all the sampling sites, the dominant bacterial phyla were (relative abundance>10%) *Proteobacteria* (43.12%), *Acidobacteria* (21.61%) and *Actinobacteria* (14.84%), and subdominant bacterial phyla (relative abundance ranging from 1% to 10%) were *Chloroflexi* (8.67%), *Firmicutes* (2.80%), *Planctomycetes* (2.19%), *Gemmatimonadetes* (1.83%) and *Verrucomicrobia* (1.05%) (Fig. 1a,c). The relative abundances of dominant phyla and subdominant phyla among elevations had no significant difference, while a norank taxon was a significant difference (Fig. 1e). On the genus level, for all samples, there were 19 taxonomic groups with a relative abundance greater than 1% (Fig. 1b). *Variibacter* (9.75%), a norank genus of *Acidobacteria* (9.37%), a norank genus of *DA111* (9.06%) and *Acidothermus* (6.30%) are most dominant genera (Fig. 1b,d). The dominant genera at different elevations were different. *Variibacter* is the most dominant genus at elevations of 2350 m and 2500 m, while a norank genus of *Acidobacteria* (9.94%) and a norank genus of *DA111* (10.80%) are the most abundant at 2050 m and 2200 m, respectively. In the 19 genera with relative abundance greater than 1%, the relative abundances of *Variibacter*, a norank genus of *Acidimicrobiales*, *Acidibacter*, a norank genus of *HSB_OF53-F07* and a norank genus of *Beijerinckiaceae* showed significant variations (*P* < 0.05) among the elevations (Fig. 1f). The relative abundances of *Variibacter* increased along the increased elevation from 2050 m to 2350 m while decreased at 2500 m (Fig. 1f).

**Figure 2:**
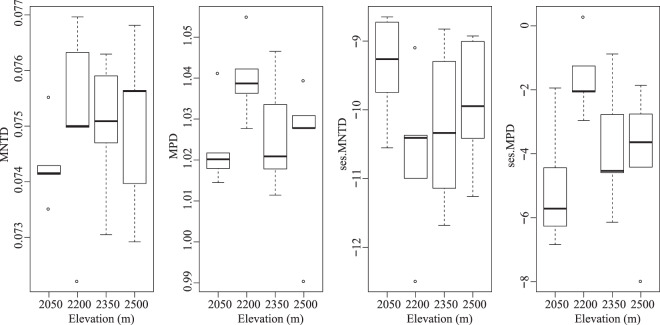
Variation of mean pairwise distance (MPD), mean nearest taxon distance (MNTD) and their standardized effect sizes (ses.MPD, ses.MNTD) of bacterial communities at different elevations.

**Figure 3:**
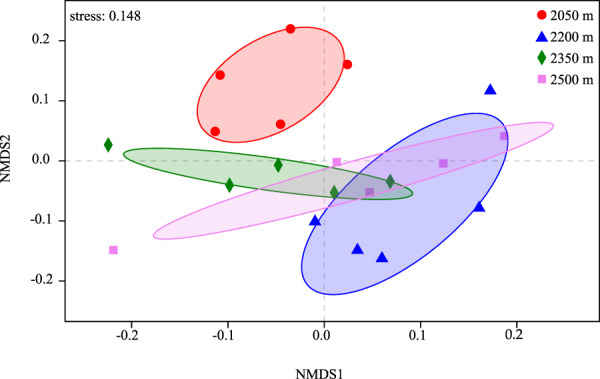
Non-metric multidimensional scaling (NMDS) ordination of microbial communities at different elevations using Bray–Curtis dissimilarity.

Based on variance analysis (One-way ANOVA) and Duncan’s multiple range test at *P* < 0.05. We found the Chao1 estimator and phylogenetic diversity (Faith’s PD) at the lowest elevation (2050 m) significantly higher than those of other elevations (*P* < 0.05, Table S2). However, the species richness and diversity indices between 2200 and 2350 m, 2350 and 2500 m were not pronounced (Table S2). Mean pairwise distance (MPD) and mean nearest taxon distance (MNTD) had no significant differences at elevations, while showed highest values at 2200 m site (Fig. 2). Both of the standardized effect sizes of MNTD and MPD (ses.MNTD and ses.MPD) values obtained using the null model were significantly negative. The similar variation of ses.MPD values at elevations is consistent with that of MPD, and the standardized metric showed a variation at elevations that was different from that of MNTD (Fig. 2). The Non-metric multidimensional scaling (NMDS) ordination showed that the members of the 2050 m site were clearly separated from the other clusters, and the difference/variation between 2050 m and 2200 m was the most highly (Fig. 3).

**Figure 4:**
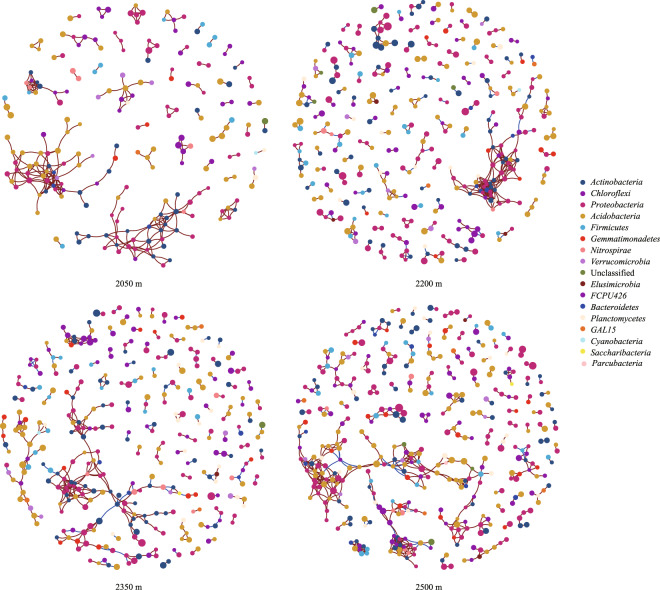
Co-occurrence network analysis of rhizosphere bacterial of ancient wild tea plants at different elevations in Qianjiazhai Reserve. The red and blue color of each connection between two nodes is positive and negative relationships of Spearman’s correlation coefficients.

According to the topological properties of the co-occurrence networks, community complexity of bacteria nadired at the 2050 m, as visible as the lowest number of nodes and edges (Fig. 4; Table S3). The average degree and the number of nodes and edges were the highest at 2500 m, and the clustering coefficient was the highest at 2200 m. These nodes belong to 17 bacteria phyla, with *Actinobacteria*, *Chloroflexi*, *Proteobacteria*, *Acidobacteria* were mainly found at sites.

### Correlation between bacterial communities and soil physicochemical properties

**Figure 5:**
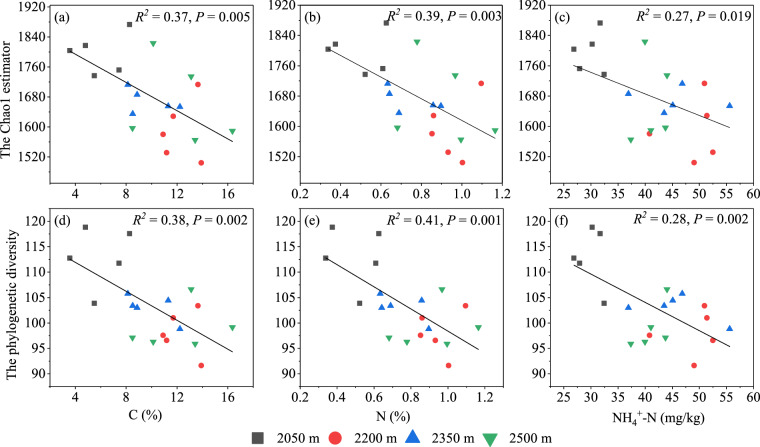
Linear relationships between Alpha diversity and physicochemical properties. (**a**) The Chao1 estimator-C%; (**b**) The Chao1 estimator-N%; (**c**) The Chao1 estimator-NH_4_^+^-N; (**d**) The phylogenetic diversity-C%; (**e**) The phylogenetic diversity-N%; (**f**) The phylogenetic diversity- NH_4_^+^-N.

Pearson correlations showed that phylogenetic diversity (Faith’s PD) and the Chao1 estimator were negatively correlated with soil N (*P* < 0.01), soil C (*P* < 0.01) and soil NH_4_^+^-N (*P* < 0.05) (Table S4). Meanwhile, we used linear regression analysis to study the relationship between the alpha diversity (Faith’s PD and the Chao1 estimator) and soil characteristics. Both the Faith’s PD and the Chao1 estimator were decreased as the increase of N, C and NH_4_^+^-N contents (Fig. 5). The stepwise multiple regression analysis showed that the ses.MPD was mainly affected by NH_4_^+^-N (*R*^2^ = 0.28, *F* = 7.00, *P* = 0.02), and the ses.MNTD was related to N and soil pH (*R*^2^ = 0.40, *F* = 5.69, *P* = 0.01; Table S5).

**Figure 6:**
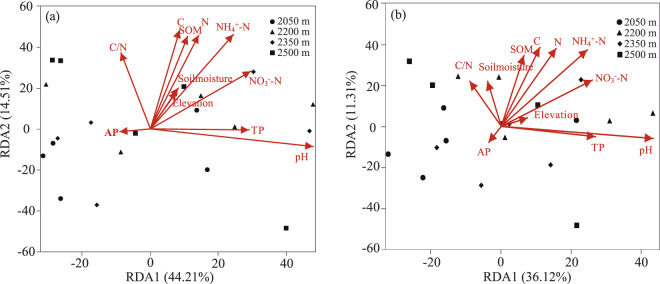
Redundancy analysis between soil characteristics and rhizobacteria. (**a**) bacterial phyla level (**b**) bacterial genera level. TP, total phosphorus; AP, available phosphorus, SOM, soil organic matter; N, total nitrogen; C, total carbon; NH_4_^+^-N, ammonium nitrogen; NO_3_^–^N, nitrate nitrogen; SM, Soil moisture; pH, soil pH.

Partial Mantel tests indicated that bacterial community composition was significantly correlation with soil NH_4_^+^-N (*r* = 0.37, *P* = 0.001), soil N (*r* = 0.35, *P* = 0.004), soil C (*r* = 0.31, *P* = 0.004) and elevations (*r* = 0.17, *P* = 0.03), while the other factors did not show a significant correlation (Table 2). According to the redundancy analysis (RDA), of all the environmental variables examined, the bacteria phyla at four elevations were mainly effected by rhizosphere soil NH_4_^+^-N (Conditional effect = *R*^2^ = 0.56, *P* = 0.001), soil pH (*R*^2^ = 0.49, *P* = 0.004), N (*R*^2^ = 0.47, *P* = 0.008), C (*R*^2^ = 0.45, *P* = 0.009), SOM (*R*^2^ = 0.34, *P* = 0.03), and NO_3_^–^N (*R*^2^ = 0.33, *P* = 0.03; Fig. 6a). For most bacterial phyla (relative abundance>1%), only one soil property variable was retained in the stepwise regression equation (Table S6). The relative abundances of *Proteobacteria* and *Acidobacteria* were significantly correlated with soil pH (*P* < 0.05). Nevertheless, for the correlation, one is positive and the other is negative. Meanwhile, *Actinobacteria* showed a definitely positive correlation with soil NO_3_^–^N (*R*^2^ = 0.39, *F* = 5.38, *P* = 0.016), and the relative abundances of *Chloroflexi* were negatively correlated with rhizosphere soil NH_4_^+^-N (*R*^2^ = 0.52, *F* = 19.69, *P* > 0.001; Table S6). On the genus level, RDA showed that NH_4_^+^-N, C, N, SOM, soil pH and NO_3_^–^N influenced bacteria in different elevations, and all bacterial with relative abundance greater than 1% could be explained by one soil properties at least (Fig. 6b, S1). Furthermore, the regression equations showed that soil total phosphorus (TP) affected the relative abundance of 6 bacterial genera, including *Variibacter* which with the highest relative abundance (Figure S1).

**Table 2:** Partial Mantel test results for the correlation between bacterial community composition and soil characteristics along the elevational gradients.

Variables	NH4+-N	C	N	TP	SOM	C/N	AP	NO_3_^–^N	Soil pH	SM
r	**0.39**	**0.33**	**0.36**	0.16	0.13	0.07	0.04	0.03	−0.02	0.02
*P*	**0.001**	**0.004**	**0.005**	0.22	0.32	0.60	0.78	0.81	0.84	0.86

TP, total phosphorus; AP, available phosphorus, SOM, soil organic matter; N, total nitrogen; C, total carbon; NH_4_^+^-N, ammonium nitrogen; NO_3_^–^N, nitrate nitrogen.

## Discussion

Plants constantly adapt and change the soil during the process of succession. In this study, the contents of most the soil physiochemical in the rhizosphere of the ancient wild tea are lowest and highest at 2050 m and 2500 m, respectively (Table 1, S1). At 2050 m, compared to other higher elevations, the ancient wild tea community was more susceptible to human activities (grazing, logging, etc.) which might be reduced litter and result in hardening of soil, further lead to a decrease in soil nutrients. However, the lowest and highest contents of NH_4_^+^-N was observed at 2050 m and 2200 m, respectively, and NO_3_^–^N also showed the highest value at 2200 m (Table 1). At different elevations, the difference of soil nitrogen process mediated by root secretions of ancient tea trees might be one of the factors causing the change of NH_4_^+^-N and NO_3_^–^N contents. NH_4_^+^-N and NO_3_^–^N were the two main nitrogen forms for tea trees absorbing and utilizing^19^, and tea plants have the characteristics of partial absorption and utilization of NH_4_^+^-N^20^. In all soil samples, the soil pH value varied from 4.56 to 4.64, which was the optimal soil pH (4.5–6.0) for tea plants. As previous studies reported, soil acidification might be caused by leaf litter and exudates of tea plants such as organic acids, carbonic acid and polyphenols^21,22^.

In this study, Illumina sequencing analyses revealed that the rhizospheric bacterial communities of the ancient wild tea were largely dominated by *Proteobacteria* (43.12%), *Acidobacteria* (21.61%) and *Actinobacteria* (14.84%) at four elevation sites, and it is similar to the previous study on tea orchard^23^. In our study, the dominance of these three phyla in the rhizosphere of ancient wild tea was further confirmed in the genera level (Fig. 1b,d; S1). However, Arafat^16^ showed that *Proteobacteria* and *Bacteroidetes* were dominant rhizospheric bacterial phylum in the 30-year-old tea plantation, which might be related to the age and environment conditions of the tea tree community^24,25^. To the best of our knowledge, the *Proteobacteria* play a major role in the global carbon, nitrogen and sulfur cycling^26^. The phylum *Proteobacteria* was the most dominant phyla in the soil bacteria obtained from various soils ranging from agricultural land and grassland to pristine forest^27^, and was the main rhizosphere bacteria of rice, *Allium tuberosum* and *Prosopis articulate*^28–30^. In addition, *Actinobacteria* was important for decomposition of organic matter to improve the quality of agricultural soil^31^, and it may prefer acidic soil as moderately acidophilic heterotrophic bacteria^32^. Variations of rhizospheric bacterial phyla of the wild ancient tea tree were not significant along the elevation gradient. However, some genera belonging to the dominant phylum, which showed significant variations (*P* < 0.05) along elevations (Fig. 1f, S1). For instance, the relative abundances of *Variibacter* increased along the increased elevation from 2050 m to 2350 m while decreased at 2500 m (Fig. 1f). The *Variibacter*, strictly aerobic, Gram-negative, was isolated from soil of the lava forest, Gotjawal, located in Aewol, Jeju, Korea^33^. It has also been reported as a dominant genus form the soil in karst, saline lake and forests from the region surrounding karst plateau lakes in Guizhou Province^34^. In our study, for the rhizospheric bacterial communities of ancient wild tea trees, the dominant phylum is similar while the dominant genera are different at different elevations in Qianjiazhai Reserve. These unique genera taxa in order to better adapt to the corresponding habitat among elevations.

According to the topological properties of the co-occurrence networks, community complexity of bacteria nadired at the 2050 m, as visible as the lowest number of nodes and edges (Fig. 4; Table S3). The complex interactions within microbial communities may contribute to the poor organic matter and insufficient nutrients in the rhizosphere of tea trees at 2050 m, which have few niches and thus with fewer opportunities for interaction between different species^35,36^. These nodes are mainly bacterial phylum with higher relative abundance, such as *Actinobacteria*, *Chloroflexi*, *Proteobacteria* and *Acidobacteria*. These results revealed that the dominant bacterial phyla in the ancient tea tree bacterial community may play critical ecological functions relating to the construction of the bacterial community^37^.

Elevation is correlated with the variables that affect the ecosystem and play a key role in influencing microbial diversity. In this study, although we found the significantly highest rhizosphere bacterial diversity (the Chao1 estimator and phylogenetic diversity) in the ancient wild tea at the lowest elevation site 2050 m, and did not found an elevational diversity gradient pattern in QianJiaZhai Reserve. As previously reported in soil bacterial studies, for example, Shen^38^ reported that soil bacterial diversity exhibited no pattern among elevational gradient on Changbai Mountain, the research of Debnath^11^ on *Rhododendron arboreum* along Eastern Himalayan Slope in Tawang came to the same conclusion. Our result is inconsistent with the findings that the unique stair-step pattern observed in Gongga Mountain^39^ or unimodal pattern in alpine tundra^40^ about soil microorganisms, the small saddle pattern of rhizospheric bacteria with *Stellera chamaejasme*^41^. A likely explanation for this differs was that the sampling sites of different elevational gradient, that is, requires broader and more fine-grained spatial extent during sampling^11,39^. On the side, inconsistent plant categories and methods of microbial community analysis may also influence the survey result^41^. Both of the standardized effect sizes of mean nearest taxon distance and mean pairwise distance (ses.MNTD & ses.MPD) values, obtained using the null model were significantly negative (ses.MNTD & ses.MPD < 0; Fig. 2), indicating that bacterial community assembly process tended to be more phylogenetically clustered than expected by chance, which suggested that environment plays a strong ecological filtering role in the process of community construction^42^. Previous studies have reported similar results^40,43^. Furthermore, the MNTD and ses. MNTD indicated that large differences in the phylogenetic clustering of bacteria between 2050 m and 2200 m and there were more assemblages that share many closely related species at 2050 m. This might be caused by the different combinations of bacterial taxa or complex elevational habitats.

We found the rhizospheric bacterial diversity was significantly correlated with rhizospheric soil N, C, and NH_4_^+^-N contents based on linear regression analysis and Pearson correlation analysis. Previously, many studies reported that soil pH is the crucial abiotic factor determining the changes in soil bacterial diversity along elevation^44–46^. Nevertheless, the lack of significant correlation between soil pH and bacterial diversity along elevations in this study may be pertaining to the narrow soil pH ranges (4.56–4.64) observed. Intriguingly, the vast majority of previous studies mentioned that soil carbon and nitrogen were two of the abiotic soil factors affecting bacterial diversity along elevations^38,39,45,47^, whereas only a few reported that soil C and N were the most prominent abiotic soil factors rather than soil pH or any other. For example, according to a prior study in alpine tundra, soil total carbon content and C:N ratio were the most prominent factors influencing bacterial diversity patterns^40^. Our findings suggested that rhizosphere bacterial alpha diversity of wild ancient tea among elevation in QianJiaZhai Reserve might be driven by the contents of rhizosphere nitrogen (Total N and NH_4_^+^-N) and total carbon. The stepwise multiple regression analysis showed that the ses.MPD was mainly affected by NH_4_^+^-N (*R*^2^ = 0.28, *F* = 7.00, *P* = 0.02), which revealed that NH_4_^+^-N could explain 28% of ses.MPD variation. The MPD, mainly reflects the deep phylogenetic structure in a phylogeny^48^, is typically thought to be more sensitive to tree-wide patterns of phylogenetic clustering or overdispersion than to the structure near the tips^49^. Furthermore, the ses.MNTD was related to N and soil pH (*R*^2^ = 0.40, *F* = 5.69, *P* = 0.01; Table S5). As such, both low soil N content and high soil pH might lead to the clustering of nearest taxon at 2050 m. Shen *et al*. also showed that one of the reasons for the phylogenetic clustering at different elevations might be closely related to sharply low nitrogen contents^40^. In our study, NMDS analyses indicated that the bacterial communities of the 2050 m site were clearly separated from the other clusters (2200, 2350 and 2500 m sites) (Fig. 3). According to the Partial Mantel test, the bacterial community structure at four elevational sites was highest associated with rhizosphere soil NH_4_^+^-N, followed by soil N, C, and elevation (Table 2). According to the redundancy analysis (RDA), NH_4_^+^-N also had the greatest impact on the classification of bacterial phylum and genera levels of the ancient wild tea plants on the elevation gradient (Fig. 6a,b). Thus, rhizospheric soil NH_4_^+^-N is the predominant abiotic soil factor in the rhizospheric bacterial community structure of ancient wild tea trees among the elevation gradient in QianJiaZhai Reserve. With respect to soil NH_4_^+^-N, which was one of the prominent abiotic soil factors that affected bacterial communities at 0–5 cm soil depth along the elevation gradients in Tibetan Plateau^50^. In another prior study, a significant correlation between soil NH_4_^+^-N and bacterial communities in rhizospheres of *Rhododendron arboretum* along Eastern Himalayan Slope in Tawang was been also found^11^. Besides NH_4_^+^-N, rhizosphere soil N, C, soil pH and SOM also play important roles in the structure of bacterial communities on elevation gradients as reported in previous studies (Table 2, Fig. 6a,b). For instance, total nitrogen was one determining factor in distributions of bacterial community at relatively high elevation of Himalayan Tawang region^11^. Both total nitrogen and carbon were significantly related to bacterial community structure along the elevational gradients of 1800–4100 m on Gongga Mountain^39^. In addition, Shen^40^ found that soil carbon and nitrogen were the most important factors in structuring bacterial communities across a small-scale elevational gradient in alpine tundra. Taken together, rhizospheric soil NH_4_^+^-N, soil N, C, soil pH and SOM appeared to have effects on bacterial community structure of ancient wild tea among elevational gradient in QianJiaZhai Reserve, with rhizospheric soil NH_4_^+^-N being the key influencing soil factor by Partial Mantel test and RDA.

For most bacterial phyla (relative abundance> 1%), only one soil property variable was retained in the stepwise regression equation (Table S6). As such, the results show that in the rhizosphere of ancient tea trees, most bacterial phyla (relative abundance> 1%) changed along elevation are driven by only a few soil properties. For instance, the relative abundances of *Proteobacteria* were positively correlated with soil pH, while *Acidobacteria* showed the opposite pattern, although only the mild fluctuations (soil pH ranged from 4.56 to 4.64), which concurred with other reports^39,51,52^. These results suggested that soil pH was considered as an important factor of the rhizosphere bacterial community composition in the ancient wild tea by affecting the dominant phylum (*Proteobacteria* and *Acidobacteria*). The relative abundance of *Actinobacteria* was related to rhizospheric soil NO_3_^–^N and NH_4_^+^-N (*R*^2^ = 0.39, *F* = 5.38, *P* = 0.02; Table S6). A similar result was reported by previous studies in an acidic forest soil^53^. In addition, both the relative abundances of *Nitrospirae* and *Chloroflexi* were negatively associated with the contents of rhizospheric soil NH_4_^+^-N. Taken together, a higher soil NH_4_^+^-N content would reduce the abundance of some taxa (e.g., *Chloroflexi*); however, copiotrophic groups (e.g., *Proteobacteria* and *Actinobacteria*) could have high richness. These findings were previously explained by the *copiotrophic* hypothesis^51^. In summary, the aforementioned result demonstrates that rhizosphere inorganic nitrogen (NH_4_^+^-N&NO_3_^–^N) and the soil pH value play a key role in rhizospheric bacterial community phyla of the ancient wild tea in QianJiaZhai Reserve.

In conclusion, the rhizosphere bacterial community of the ancient wild tea tree is unique in diversity, phylogeny and bacterial network structure at 2050 m site, which is attributed to the interaction of multiple soil factors, especially ammonium nitrogen. The NH_4_^+^-N is a key driving soil factor affecting the rhizosphere bacterial community of wild ancient tea trees on the elevation gradients, as well as the relative abundance of some specific bacterial phyla/genera such as *Proteobacteria*, *Actinobacteria*, *Chloroflexi*, *Bryobacter* and *Acidibacter*. Our study is the first time to examine the rhizospheric bacterial community of such ancient wild tea tree (DBH approximately 0.3 m, with probably more than 1000 years old), which is the dominant vegetation species in QianJiaZhai Reserve. Hence, our findings will provide researchers the necessary basic information to better understanding the ecological adaptation of ancient wild tea trees. In future, more research needs to be focused on integrative studies in soil microbiology, to reveal the “rhizosphere effect” of ancient wild tea plants.

## Materials and Methods

### Site selection and soil sampling

The QianJiaZhai Reserve (101°14′E; 24°17′N) is located in Yun-nan Ailao Mountains (Zhen-yuan County), Southwest of China. The annual average temperature here is 10–12 °C, and the precipitation is more than 1500 mm. The frost period is from November to March the next year, and the snow period is from December to February the next year. The terrain in the region is complicated, and there are ancient wild tea trees along the elevational gradients (2100–2500 m). As the dominant species in the region, the diameter at breast height of wild ancient tea was frequently more than 0.3 m (ca. 1000 years old). Besides the wild ancient tea, the subdominant species are *Lithocarpus xylocarpus* and *Manglietia insignis* (Table S7)^54^.

We set four sites to cover the entire elevation range of wild ancient tea: 2050 m, 2200 m, 2350 m, and 2500 m (Table S7). Five sampling plots were randomly chosen within each elevation along transects of 150 m length. At each sampling plot, mixed rhizospheric soil samples (according to Debnath^11^) were taken from three randomly selected tea plants (the diameter at breast height was about 0.3 m) and separated by at least 5 m from the others. In total, 20 samples were collected and placed in labeled sterile polypropylene bags in an icebox and transported to the laboratory for analysis. Upon arrival, one part of soil samples is spread out on clean paper to dry in a ventilated room and storage for subsequent element analysis. Another part stored at −80 °C for assessing the rhizospheric bacterial communities.

### Physico-chemical characteristics of soil

All rhizospheric soil subsamples were naturally air-dried at indoor and subsequent analysis was performed after screening with a 2 mm sieve. Methods for measuring soil pH with a pH meter: mixing the soil sample and KCl solution (1:2.5 wt/vol) after vigorous shaking and suspending for 1 h. Soil moisture determined was gravimetrical. For the determination of total carbon and total nitrogen the concentrations, the elemental analyzer (Vario MAX, Elementar, Germany) was used. The NO_3_^−^-N and NH_4_^+^-N were measured according to standard methods^55^. Other soil chemicals: available phosphorus^56^, and organic matter content^57^ were also determined.

### DNA Extraction, PCR amplification, and MiSeq sequencing

Rhizospheric soil genomic DNA was extracted in triplicate from fresh soil with FastDNA® Spin kit (MP Biomedicals, Santa Ana, CA, USA) as specified in operating instruction. For the extracted soil DNA, its concentration and quality were assessed by the NanoDrop ND-2000 Spectrophotometer (NanoDrop Technologies, Delaware, USA). Once the DNA quality assessment was completed, it was promptly stored at −20 °C until use. The V3-V4 region of the bacterial 16 S rRNA genes were amplified using conventional primers 338 F (5′-ACTCCTACGGGAGGCAGCAG-3′) and 806 R (5′-GGACTACHVGGGTWTCTAAT-3′). PCR was performed in 20 µL of reaction mixture were set up as follows: 5 μL of 5×Fastfu buffer, 2 µL of dNTPs (2.5 mM), 0.8 µL of forward and reverse primers (5 µM), 0.4 µL of TransStart Fastpfu DNA Polymerase, 0.2 µL of bovine serum albumin, 10 ng of template DNA, and add double distilled water (ddH_2_O) to 20 µL. Using an Applied Biosystems GeneAmp® 9700 (Applied Biosystems, USA) with the following PCR conditions: 3 minutes at 95 °C, 30 seconds at 95 °C, annealing at 30 seconds for 55 °C, 45 seconds at 72 °C, 10 minutes at 72 °C, 10 °C until halted by user. An equal amount of PCR products was pooled together in a single tube and run on an Illumina MiSeq PE300 platform (Illumina, San Diego, CA, USA).

### Processing of illumina sequencing data

Paired-end reads were merged by the FLASH software^58^. Sequences obtained by Illumina Miseq sequencing were processed and analyzed with the Quantitative Insights into Microbial Ecology pipeline (QIIME)^59^. Using the USEARCH algorithm to identified and removed Chimeric sequences^60^. Simultaneously, in order to rectify the influence of widely different sequencing depth, we rarefied entire samples to the equally sequencing depth by stochastic chosing subset of 25890 sequences from each soil sample. The sequences obtained across samples were specified as operational taxonomic units (OTUs) at 97% analogy. All representative sequences were classified taxonomically according to Silva database.

### Diversity and statistical analysis

The Chao1-richness^61^ and Faith’s phylogenetic diversity (PD)^62^ were calculated by with the Mother software (v.1.30.1) to estimate the bacterial alpha diversity. The function ses.mpd and ses.mntd were used to calculate mean pairwise distance (MPD) and mean nearest taxon distance (MNTD)^49^, respectively. We used a network analysis method to visualize the co-occurrence of community structure, characterize intra-community interactions and identify potential shared niches^37^. Non-metric multidimensional scaling analysis (NMDS) tests of Bray-Curtis similarity distance values at the OTU level was used to observe if the samples at different elevational sites formed unique phylogenetically related clusters. Linear regression analysis and Pearson correlation analyses were employed to determine the relationships between abiotic rhizospheric factors and alpha diversity. Partial Mantel based on Bray-Curtis similarities were used to depict the relationships between bacterial community structure and rhizospheric soil factors with Qiime software, while the redundancy analysis (RDA) were carried to further reveal rhizospheric soil elements contribution degree to bacterial at the four sites using CANOCO version 5. In addition, before the RDA analysis, a detrended correspondence analysis for the specific microbial groups was performed to confirm that the linear ordination method was appropriate for the analyses (gradient lengths<3). Used stepwise multiple regression to find the most important some factors related to MPD, MNTD and the relative abundance of bacteria classified at the phyla and genus levels.

## Supplementary information

Supplementary Information.

## Data Availability

Illumina MiSeq amplicon sequences of 16 S rRNA genes of bacteria are available in the NCBI Sequence Read Archive (www.ncbi.nlm.nih.gov/sra) as BioProject PRJNA629666.

## References

[1] Fangci, Z., Fuliang, Y. & Zhang, S. Opinions on the investigation and study of ancient wild tea trees in the QianJiaZhai Reserve of Yun-nan Ailao Mountains (Zhen-yuan County). Agricultural Archaeology. 216–217 (1997).

[2] Lu H, Min Q, Yuan Z (2011). Resources, Value and agricultural heritage characteristics of the ancient tea plant in the middle and lower reaches of the Lancang River. Resources. Science..

[3] Philippot L, Raaijmakers JM, Lemanceau P, van der Putten WH (2013). Going back to the roots: the microbial ecology of the rhizosphere. Nat Rev Microbiol..

[4] Castrillo G (2017). Root microbiota drive direct integration of phosphate stress and immunity. Nature..

[5] Staley C (2017). Diurnal cycling of rhizosphere bacterial communities is associated with shifts in carbon metabolism. Microbiome..

[6] Berendsen RL, Pieterse CM, Bakker PA (2012). The rhizosphere microbiome and plant health. Trends Plant Sci..

[7] Larousse M (2017). Tomato root microbiota and Phytophthora parasitica-associated disease. Microbiome..

[8] Hubbard CJ (2019). The effect of rhizosphere microbes outweighs host plant genetics in reducing insect herbivory. Mol Ecol..

[9] Bertin C, Yang X, Weston LA (2003). The role of root exudates and allelochemicals in the rhizosphere. Plant Soil..

[10] Bais HP, Weir TL, Perry LG, Gilroy S, Vivanco JM (2006). The role of root exudates in rhizosphere interactions with plants and other organisms. Annu Rev Plant Biol..

[11] Debnath R (2016). Rhizospheric bacterial community of endemic Rhododendron arboreum Sm. Ssp. delavayi along eastern Himalayan slope in Tawang. Front Plant Sci..

[12] Zhao W, Qi X, Lyu J, Yu Z, Chen X (2015). Characterization of microbial community structure in rhizosphere soils of Cowskin Azalea (Rhododendron aureum Georgi) on northern slope of Changbai Mountains, China. Chinese Geographical Science..

[13] Pandey A, Palni LMS (1997). Bacillus species: The dominant bacteria of the rhizosphere of established tea bushes. Microbiological Research..

[14] Kaiyang L (2016). Bacteria distribution diversity in tea rhizospheric soil from different habitats at Nannuo Mountain based on 16S rRNA sequence analysis. Journal of Kunming University of Science and Technology..

[15] Arafat, Y. *et al*. Spatial distribution patterns of root-associated bacterial communities mediated by root exudates in different aged ratooning tea monoculture systems. Int J Mol Sci. 18 (2017). 10.3390/ijms18081727 PMC5578117 28786955

[16] Li Y (2017). Characterizing rhizosphere microbial communities in long-term monoculture tea orchards by fatty acid profiles and substrate utilization. European Journal of Soil Biology..

[17] Tan L (2019). Responses of microbial communities and interaction networks to different management practices in tea plantation soils. Sustainability..

[18] Huang X, Tang T, Jiang Y, Feng C, Cheng X (2015). Genetic diversity of wild tea plant in different altitude in Qianjiazhai. Journal of Tea Science..

[19] Oh K, Kato T, Xu H-lian (2008). Transport of nitrogen assimilation in xylem vessels of green tea plants fed with NH4+-N and NO3–N. Pedosphere..

[20] Ruan L (2016). Characteristics of NH4+ and NO3- fluxes in tea (Camellia sinensis) roots measured by scanning ion-selective electrode technique. Scientific Reports..

[21] Pandey., A. & L.M.S.Palni. The rhizosphere effect of tea on soil microbes in a Himalayan monsoonal location. Biol Fert Soils. 131-137 (1996).

[22] Li S (2016). Rates of soil acidification in tea plantations and possible causes. Agriculture, Ecosystems & Environment..

[23] Li YC (2016). Variations of rhizosphere bacterial communities in tea (Camellia sinensis L.) continuous cropping soil by high-throughput pyrosequencing approach. Journal of applied microbiology..

[24] Watt M, Hugenholtz P, White R, Vinall K (2006). Numbers and locations of native bacteria on field-grown wheat roots quantified by fluorescence *in situ* hybridization (FISH). Environ Microbiol..

[25] Li J (2018). Variation of soil bacterial communities along a chronosequence of Eucalyptus plantation. PeerJ..

[26] Kersters, K. *et al*. Introduction to the Proteobacteria. (2006).

[27] Janssen PH (2006). Identifying the dominant soil bacterial taxa in libraries of 16S rRNA and 16S rRNA genes. Appl Environ Microbiol..

[28] Lu Y, Rosencrantz D, Liesack W, Conrad R (2010). Structure and activity of bacterial community inhabiting rice roots and the rhizosphere. Environmental Microbiology..

[29] Qin, X. *et al*. Changes in Soil microbial community structure and functional diversity in the rhizosphere surrounding tea and soybean. Journal of Agricultural Sciences. 12 (2017).

[30] Galaviz C (2018). Root growth improvement of mesquite seedlings and bacterial rhizosphere and soil community changes are induced by inoculation with plant growth-promoting bacteria and promote restoration of eroded desert soil. Land Degradation & Development..

[31] Strap, J. L. in Bacteria in agrobiology: plant growth responses.285-307 (Springer (2011).

[32] Lin YT (2010). Bacterial community diversity in undisturbed perhumid montane forest soils in Taiwan. Microb Ecol..

[33] Kim KK (2014). Variibacter gotjawalensis gen. nov., sp. nov., isolated from soil of a lava forest. Antonie Van Leeuwenhoek..

[34] Deng Y, Yu L, Zhang Y (2019). Bacterial diversity in the soil surrounding the karst plateau lakes in Guizhou. Biotic Resources..

[35] Jiang Y, Song H, Lei Y, Korpelainen H, Li C (2019). Distinct co-occurrence patterns and driving forces of rare and abundant bacterial subcommunities following a glacial retreat in the eastern Tibetan Plateau. Biology and Fertility of Soils..

[36] Sun HY, Wu YH, Zhou J, Bing HJ (2016). Variations of bacterial and fungal communities along a primary successional chronosequence in the Hailuogou glacier retreat area (Gongga Mountain, SW China). Journal of Mountain Science..

[37] Jiang Y (2018). Divergent assemblage patterns and driving forces for bacterial and fungal communities along a glacier forefield chronosequence. Soil Biology and Biochemistry..

[38] Shen C (2013). Soil pH drives the spatial distribution of bacterial communities along elevation on Changbai Mountain. Soil Biology and Biochemistry..

[39] Li J (2018). Stair-step pattern of soil bacterial diversity mainly driven by pH and vegetation types along the elevational gradients of Gongga Mountain, China. Front Microbiol..

[40] Shen C, Ni Y, Liang W, Wang J, Chu H (2015). Distinct soil bacterial communities along a small-scale elevational gradient in alpine tundra. Front Microbiol..

[41] Ni Y, Yang T, Zhang K, Shen C, Chu H (2018). Fungal Communities along a small-scale elevational gradient in an Alpine Tundra are determined by soil carbon nitrogen ratios. Front Microbiol..

[42] Chu H (2016). Bacterial community dissimilarity between the surface and subsurface soils equals horizontal differences over several kilometers in the western Tibetan Plateau. Environmental Microbiology..

[43] Wang J, Soininen J, He J, Shen J (2012). Phylogenetic clustering increases with elevation for microbes. Environ Microbiol Rep..

[44] Xia Z (2016). Biogeographic distribution patterns of bacteria in typical Chinese forest soils. Front Microbiol..

[45] Ren B (2018). Soil pH and plant diversity shape soil bacterial community structure in the active layer across the latitudinal gradients in continuous permafrost region of Northeastern China. Sci Rep..

[46] Zhang J (2019). Variations in pH significantly affect cadmium uptake in grafted muskmelon (Cucumis melo L.) plants and drive the diversity of bacterial communities in a seedling substrate. Plant Physiol Biochem..

[47] Gutierrez-Ruacho, O. *et al*. Abundance of rhizospheric bacteria and fungi associated with Fouquieria columnaris at Punta Cirio, Sonora, Mexico. Revista Mexicana de Biodiversidad. 89 (2018).

[48] Chun JH, Lee CB (2018). Partitioning the regional and local drivers of phylogenetic and functional diversity along temperate elevational gradients on an East Asian peninsula. Sci Rep..

[49] Webb CO, Ackerly DD, McPeek MA, Donoghue MJ (2002). Phylogenies and community ecology. Annual Review of Ecology Systematics.

[50] Yuan Y, Si G, Wang J, Luo T, Zhang G (2014). Bacterial community in alpine grasslands along an altitudinal gradient on the Tibetan Plateau. FEMS Microbiol Ecol..

[51] Fierer N (2012). Comparative metagenomic, phylogenetic and physiological analyses of soil microbial communities across nitrogen gradients. The ISME Journal..

[52] Shen, C. *et al*. Soil pH dominates elevational diversity pattern for bacteria in high elevation alkaline soils on the Tibetan Plateau. FEMS Microbiol Ecol. 95 (2019). 10.1093/femsec/fiz003 30629166

[53] Nie Y (2018). Ammonium nitrogen content is a dominant predictor of bacterial community composition in an acidic forest soil with exogenous nitrogen enrichment. Sci Total Environ..

[54] Yong C, Hua Z, Guang-tao M, Ji-pu S, Guo-ping Y (2011). Population structure and distribution pattern of dominant Tree species in ancient tea tree community in Ailao Mountains of Yunnan Province. China. Forest Research..

[55] Kou L (2018). Simulated nitrogen deposition affects stoichiometry of multiple elements in resource-acquiring plant organs in a seasonally dry subtropical forest. Sci Total Environ..

[56] Olsen, S. & Sommers, L. Phosphorus methods of soil analysis. Chemical and Microbiological Properties. 581–893 (1982).

[57] Maia, C., Novotny, E., Rittl, T. & Hayes, M. H. B. Soil organic matter: chemical and physical characteristics and analytical methods. A Review. Current Organic Chemistry Current Organic Chemistry. 2985 (2013).

[58] Magoc T, Salzberg SL (2011). FLASH: fast length adjustment of short reads to improve genome assemblies. Bioinformatics (Oxford)..

[59] Caporaso J (2012). Ultra-high-throughput microbial community analysis on the Illumina HiSeq and MiSeq platforms Open. The. ISME Journal..

[60] Edgar RC, Haas BJ, Clemente JC, Quince C, Knight R (2011). UCHIME improves sensitivity and speed of chimera detection. Bioinformatics..

[61] Chao A, Shen T-J, Hwang W-H (2006). Application of Laplace’s boundary-mode approximations to estimate species and shared species richness. Australian & New Zealand Journal of Statistics..

[62] Faith DP (1992). Conservation evaluation and phylogenetic diversity. Biological Conservation..

