# Supplementary material for: Change of rhizospheric bacterial community of the ancient wild tea along elevational gradients in Ailao mountain, China
Source: Sci Rep. 2020 Jun 8;10:9203. doi: 10.1038/s41598-020-66173-9 (PMC7280300; doi:10.1038/s41598-020-66173-9)
Supplement: Supplementary file 1 — Supplementary Information. [file 41598_2020_66173_MOESM1_ESM.pdf]

Change of rhizospheric bacterial community of the ancient wild tea along elevational gradients in Ailao mountain, China

Haiyun Zi[1], Yonglei Jiang[2], Xiaomao Cheng[1], Wanting Li[1], Xiaoxia Huang[1]*

[1] Southwest Landscape Architecture Engineering Research Center of State Forestry and Grassland Administration, College of Landscape Architecture and Horticulture, Southwest Forestry University, Yunnan Kunming 650224, China

[2] Yunnan Academy of Tobacco Agricultural Sciences, Kunming, China 650021;

* Correspondence: huangxx@swfu.edu.cn

	Elevation	TP	AP	SOM	N	C	C/N	NH_4^+-N	NO_3^--N	SM
TP	0.68**									
AP	0.62**	0.61**								
SOM	0.61**	0.52*	0.31							
N	0.54*	0.51*	0.24	0.89**						
C	0.58**	0.48*	0.24	0.90**	0.99**					
C/N	0.54*	0.32	0.18	0.66**	0.61**	0.72**				
NH^{4+}-N	0.33	0.32	0.19	0.55*	0.76**	0.75**	0.50*			
NO^{3-}-N	0.20	0.61**	0.21	0.48*	0.53*	0.47*	0.14	0.43		
SM	0.10	0.14	-0.06	0.27	0.34	0.34	0.39	0.42	0.08	
pH	0.24	0.56**	0.15	0.08	0.14	0.09	-0.09	0.13	0.40	-0.02

Table S1. Pearson correlation coefficients among physiochemical properties and elevations.TP, total phosphorus; AP, available phosphorus; SOM, soil organic matter; N, total nitrogen; C, total carbon; NH^{4+}-N, ammonium nitrogen; NO^{3-}-N, nitrate nitrogen; pH, Soil pH; SM, Soil moisture. "*" means $P < 0.05$, "**" means $P < 0.01$.

Elevation	Estimators	
	Chao1	Faith's PD
2050 m	1796.5794 ± 24.3254a	112.9442 ± 2.6488a
2200 m	1591.4597 ± 37.1227b	98.0298 ± 2.0049b
2350 m	1668.6002 ± 13.7535b	103.0617 ± 1.1674b
2500 m	1662.0876 ± 50.1258b	103.0364 ± 3.8073b
ANOVA (*F*)	6.245**	5.809**

Table S2. The Chao1 estimator and phylogenetic diversity (Faith's PD) among different elevations. Based on variance analysis (One-way ANOVA) and Duncan's multiple range test; Data are indicated as mean ± standard error, n=5; The lower-case letters 'a' and 'b' indicate contrasts that are significantly at $P < 0.05$. "**" mean $P < 0.01$.

Properties	2050 m	2200 m	2350 m	2500 m
Nodes	213	365	368	428
Edges	307	454	420	633
Pos/Neg edges	307/0	404/50	348/72	565/68
Average degree	2.88	2.49	2.28	2.96
Cluster coefficient	0.48	0.61	0.51	0.54

Table S3. Bacterial network properties in the rhizosphere of ancient wild tea plants at different elevations of the Qianjiazhai Reserve.

Indexes	Elevation	TP	AP	SOM	NH_4^+-N	NO_3^--N	N	C	C/N	SM	pH
Chao1	-0.36	-0.26	-0.02	-0.41	**-0.52***	-0.24	**-0.63****	**-0.61****	-0.31	0.17	-0.07
Faith's PD	-0.37	-0.25	-0.01	-0.42	**-0.53***	-0.21	**-0.64****	**-0.61****	-0.27	0.09	-0.08

Table S4. Pearson correlations (r) between bacterial diversity and soil characteristics in the rhizosphere of ancient wild tea plants. Values in bold show the statistically significant difference (P<0.05). *: P<0.05, **: P<0.01. TP, total phosphorus; AP, available phosphorus, SOM, soil organic matter; NH_4^+-N, ammonium nitrogen; NO_3^--N, nitrate nitrogen. N, total nitrogen; C, total carbon; pH, Soil pH; SM, Soil moisture.

Indexes	Standardized regression equation	R^2	*F*	*P*
ses.MPD	$y=0.529NH_4^+$	0.28	7.003	0.016
ses.MNTD	y=-0.558N+0.388pH	0.40	5.688	0.013
MPD	null			
MNTD	null			

Table S5. Stepwise multiple regression analysis with mean pairwise distance (MPD), mean nearest taxon distance (MNTD) and their standardized effect sizes (ses.MPD, ses.MNTD). "null" mean has no regression relationship under the condition of $P<0.05$. NH_4^+, ammonium nitrogen; NO_3^-, nitrate nitrogen. N, total nitrogen; SOM, soil organic matter; SM, Soil moisture; pH, Soil pH.

Taxa	Regression equation	R^2	*F*	*P*
Proteobacteria	y=-62.724+28.139pH+0.107NH_4^+	0.37	5.026	0.019
Acidobacteria	y=100-21.879pH	0.26	6.283	0.022
Actinobacteria	y=7.247+0.273NO_3^-+0.071NH_4^+	0.39	5.378	0.016
Chloroflexi	y=17.558-0.217NH_4^+	0.52	19.690	>0.001
Firmicutes	null			
Planctomycetes	y=14.479-3.416pH	0.23	5.315	0.033
Gemmatimonadetes	null			
Verrucomicrobia	y=1.896-0.611TP	0.37	10.724	0.004
Nitrospirae	y=1.233-0.053NH_4^+ +8.475AP	0.46	7.210	0.005

Table S6. Relationships between bacterial phyla and soil characteristics that were modeled using stepwise multiple regression. "null" mean has no regression relationship under the condition of P<0.05. NH_4^+, ammonium nitrogen; NO_3^-, nitrate nitrogen; pH, Soil pH; TP, total phosphorus; AP, available phosphorus.

Elevation (m)	Coordinates	Most common family of tree/shrub/grass	Sites basic situation
2050	N 24° 27′ 83.7″ E 101° 26′ 37.1″	The ancient tea trees, *Lithocarpus xylocarpus* / Saplings of arbor / Pteridophytes	Located near the Eco Station (stream), most vulnerable to human activity
2200	N 24° 27′ 49.3″ E 101°27′ 24.2″	The ancient tea trees, *Lithocarpus xylocarpus*, *Manglietia insignis* / *Fargesia wuliangshanensis*, Saplings of arbor / Pteridophytes	Located near "the no.2 tea tree", less influenced by human activities (picking, grazing, etc.)
2350	N 24° 28′ 92.6″ E 101° 26′ 11.8″	The ancient tea trees, *Lithocarpus xylocarpus*, / *Fargesia wuliangshanensis*, *Blastus cochinchinensis* / *Pilea gracilis*	Located near the stream and path, the arbor density is small, the crown density is small and the grazing is frequent
2500	N 24° 29′ 47.9″ E 101° 26′ 24.7″	The ancient tea trees, *Lithocarpus xylocarpus*, *Cyclobalanopsis stewardiana* / Saplings of arbor / Pteridophytes, *Fordiophyton longipes*	Located near "the oldest tea tree', the protection is more comprehensive, generally there is no grazing or cutting

Table S7. Main details of the investigated sites; asl above sea level.

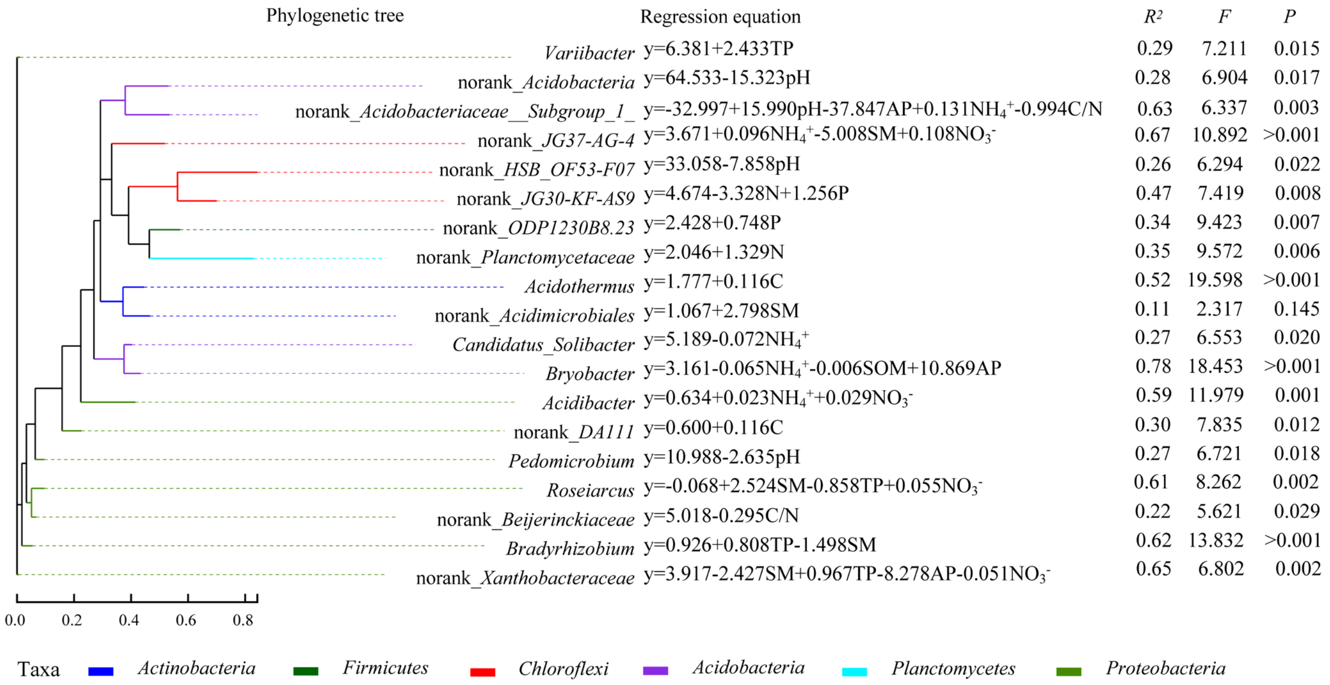

Figure S1. Relationships between bacterial (genus level) and soil characteristics that were modeled using stepwise multiple regression. TP, total phosphorus; AP, available phosphorus; SOM, soil organic matter; N, total nitrogen; C, total carbon; NH^{4+}-N, ammonium nitrogen; NO^{3-}-N, nitrate nitrogen; pH, Soil pH; SM, Soil moisture.
